# Telephone CPR Instructions in Emergency Dispatch Systems: Qualitative Survey of 911 Call Centers

**DOI:** 10.5811/westjem.2015.6.26058

**Published:** 2015-10-20

**Authors:** John Sutter, Micah Panczyk, Daniel W. Spaite, Jose Maria E. Ferrer, Jason Roosa, Christian Dameff, Blake Langlais, Ryan A. Murphy, Bentley J. Bobrow

**Affiliations:** * Arizona Department of Health Services, Bureau of EMS and Trauma System, Phoenix, Arizona; † University of Arizona, Department of Emergency Medicine, Arizona Emergency Medicine Research Center, Phoenix, Arizona; ‡ American Heart Association; § Lutheran Medical Center, Wheat Ridge, Colorado; ¶ University of Arizona College of Medicine – Phoenix, Phoenix, Arizona

## Abstract

**Introduction:**

Out-of-hospital cardiac arrest (OHCA) is a leading cause of death. The 2010 American Heart Association Emergency Cardiovascular Care (ECC) Guidelines recognize emergency dispatch as an integral component of emergency medical service response to OHCA and call for all dispatchers to be trained to provide telephone cardiopulmonary resuscitation (T-CPR) pre-arrival instructions. To begin to measure and improve this critical intervention, this study describes a nationwide survey of public safety answering points (PSAPs) focusing on the current practices and resources available to provide T-CPR to callers with the overall goal of improving survival from OHCA.

**Methods:**

We conducted this survey in 2010, identifying 5,686 PSAPs; 3,555 had valid e-mail addresses and were contacted. Each received a preliminary e-mail announcing the survey, an e-mail with a link to the survey, and up to three follow-up e-mails for non-responders. The survey contained 23 primary questions with sub-questions depending on the response selected.

**Results:**

Of the 5,686 identified PSAPs in the United States, 3,555 (63%) received the survey, with 1,924/3,555 (54%) responding. Nearly all were public agencies (n=1,888, 98%). Eight hundred seventy-eight (46%) responding agencies reported that they provide no instructions for medical emergencies, and 273 (14%) reported that they are unable to transfer callers to another facility to provide T-CPR. Of the 1,924 respondents, 975 (51%) reported that they provide pre-arrival instructions for OHCA: 67 (3%) provide compression-only CPR instructions, 699 (36%) reported traditional CPR instructions (chest compressions with rescue breathing), 166 (9%) reported some other instructions incorporating ventilations and compressions, and 92 (5%) did not specify the type of instructions provided. A validation follow up showed no substantial difference in the provision of instructions for OHCA by non-responders to the survey.

**Conclusion:**

This is the first large-scale, nationwide assessment of the practices of PSAPs in the United States regarding T-CPR for OHCA. These data showing that nearly half of the nation’s PSAPs do not provide T-CPR for OHCA, and very few PSAPs provide compression-only instructions, suggest that there is significant potential to improve the implementation of this critical link in the chain of survival for OHCA.

## INTRODUCTION

Out-of-hospital cardiac arrest (OHCA) is a leading cause of death in the United States with a survival rate of less than 8%.^1^,^2^ The American Heart Association (AHA) has promulgated the “Chain of Survival” as a framework for the successful resuscitation of victims of OHCA.^3^ The timing and quality of care provided in the first link of the “Chain” (immediate recognition and early bystander cardiopulmonary resuscitation [CPR]) is strongly associated with improved survival from cardiac arrest, yet bystander CPR is performed in less than one-half of all OHCAs.^3^–^6^ Telephone-CPR (T-CPR) is the delivery of compression and/or ventilation instructions to callers of suspected OHCA cases. T-CPR has been recognized as an integral component of an emergency medical system response to OHCA and holds enormous potential to increase bystander response and thus survival from cardiac arrest.^7^ Guidelines call for all dispatchers to be appropriately trained to provide T-CPR instructions and have an ongoing quality improvement mechanism to assure that all unresponsive adults who are not breathing normally receive appropriate T-CPR instructions as early as possible.^8^,^9^

A Public Safety Answering Point (PSAP) is a call center responsible for answering calls to an emergency telephone number for police, firefighting, and ambulance services. The purpose of this study is to describe current practices and resources available at the 9-1-1 call centers to provide T-CPR instructions to callers of OHCA events in the U.S.

## METHODS

A survey of public safety answering points (PSAPs) in the U.S. and Canada was commissioned by the Emergency Cardiac Care Committee of the AHA to determine the availability of T-CPR instructions for medical emergencies. It was estimated that there are approximately 7,000 PSAP call centers that receive 9-1-1 emergency calls for law enforcement, fire, or medical emergencies in the United States, and this report focuses on the U.S. component of the survey.

We conducted an initial pilot survey of 391 PSAPs in five states (Colorado, Georgia, Iowa, Maryland, and Oregon). These were selected from a group of 11 states for which a complete list of PSAP e-mail addresses was readily available in order to determine feasibility of the online survey instrument and to study how the survey questions functioned. We contacted these PSAPs by e-mail with a message announcing the survey, a message linking to the survey itself, and up to three reminder e-mails for non-responders. The response rates, responder comments, and times to complete the online survey were collected and used to modify questions in the final survey. Modifications were limited to changes in the response options available for five of the 23 survey questions and rephrasing of one question for clarity. The pilot survey and the modifications that were made to the national survey are available as supplementary material (Supplement 1, Pilot Survey and Modifications to National Survey).

**Figure 1:**
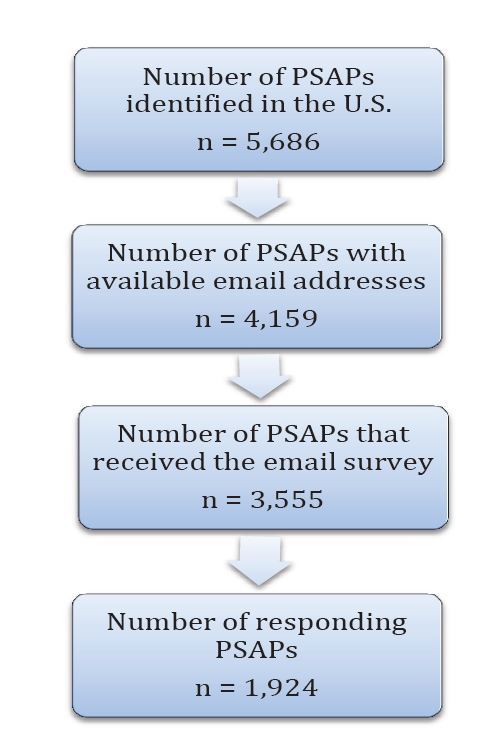
Number of responding public safety answering points in the United States. *PSAP,* public safety answering point

The final survey was conducted in the spring of 2010 and included all 50 states and the District of Columbia. We used a sequential strategy to identify PSAPs including contacting state officials, searching sheriff and police department websites, and then calling individual agencies. We identified 5,686 PSAPs; 4,159 (73%) had available e-mail addresses, and 3,555 (85%) of these e-mails were deliverable (Figure 1). Agencies were contacted in the same manner as in the pilot survey described above: a preliminary e-mail announcing the survey with a statement of endorsement from the lead state EMS official (when available) and options for completing the survey via mail or fax, an e-mail with a link to the survey, and up to three follow-up e-mails for non-responders. Messages were separated by two business days. We obtained institutional review board approval by SCL Health, Denver, CO, for this study.

### Validation

After the completion of the survey, we conducted a follow-up study by telephone to compare responding and non-responding agencies. A random sample of 51 non-responding agencies located within the five states with the lowest response rate (Illinois, Nebraska, New Jersey, South Dakota, Minnesota, with a 20%–26% response rate) and a random sample of 50 non-responding agencies located in the middle five responding states (New Mexico, New York, South Carolina, Kansas, and Virginia, with a 40%–44% response rate) were contacted. These agencies were asked to answer a truncated five-question version of the survey over the phone. We then compared responses from these non-responding agencies to those of responding agencies.

### Statistical Methods

Proportions were compared using normal approximations of the binomial distribution and Fisher’s exact method. We used one- and two-sided hypotheses at the 0.05 significance level. Means are reported with standard deviations (SD) and medians with interquartile ranges (Q1–Q3). Descriptive statistics are also reported. Analyses were performed in SAS Software 9.4 (SAS Institute, Cary NC) and R 3.1.3.^10^

## RESULTS

### Response Rate

Of the 5,686 identified PSAPs in the U.S., 3,555 (63%) received the e-mail survey. Of these, 1,924/3,555 (54%) responded to the survey (Figure 1). This response rate represents 34% (1,924/5,686) of the total number of identified PSAPs, 46% (1,924/4,159) of PSAPs with available e-mail addresses. Responding agencies represented all 50 states and the District of Columbia. Responses to selected question items reflecting the characteristics of PSAPs and their provision of T-CPR instructions are discussed at length below.

### PSAP Characteristics

The vast majority of PSAPs were public agencies (n=1,888, 98%) versus privately owned (n=20, 1%). In large part, surveys were completed by management personnel at the individual PSAPs (n=1,658, 86%). Additionally, surveys were completed by law enforcement officers (n=115, 6%), dispatch personnel (n=91, 5%), and others including 9-1-1 coordinators (n=57, 3%). Table 1a and 1b shows the breakdown of PSAPs by administrative type. PSAPs were staffed by a median number of 10 dispatchers with an interquartile range (IQR) of 6 to 16. PSAPs reported handling a median of 12,000 9-1-1 calls annually with a median of 30% of calls resulting in EMS dispatch. Among respondents, 1,199 (62%) facilities identified as primary PSAPs (9-1-1 calls arrive directly), 51 (3%) identified as secondary PSAPs (9-1-1 calls are routed from a primary PSAP), and 659 (34%) identified as both (Table 1a and 1b).

### Pre-arrival Instructions

**Figure 2:**
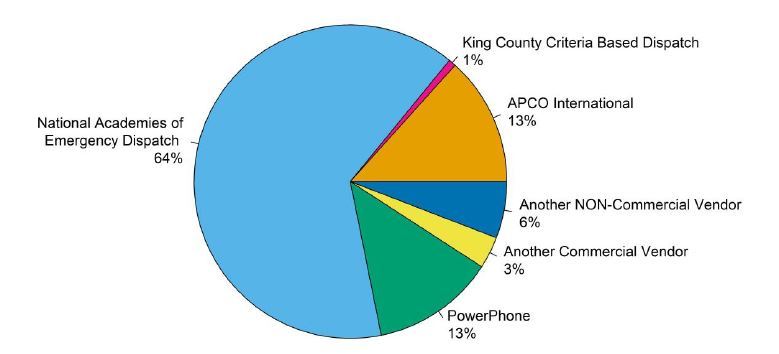
Script or guideline use by producing agency. *APCO,* association of public-safety communications officials

Of 1,924 respondents, 1,021 (53%) PSAPs reportedly provide instructions for medical emergencies. On average, 87.65% (SD 29.47%) of the call-takers who provide T-CPR instructions are certified as emergency medical dispatch dispatchers (for example, national academy of emergency dispatch [NAED] or association of public-safety communications officials [APCO] certified or another certification), and a further 7.76% (SD 23.43%) are trained, but not certified. A structured script is used by 83% of agencies providing T-CPR instructions, while 14% use only written guidelines, and 3% do not use guidelines or a script (Table 2). Of those agencies using a script or guidelines, the type of script or guidelines used for T-CPR instructions varies between PSAPs as shown in Figure 2.

**Figure 3:**
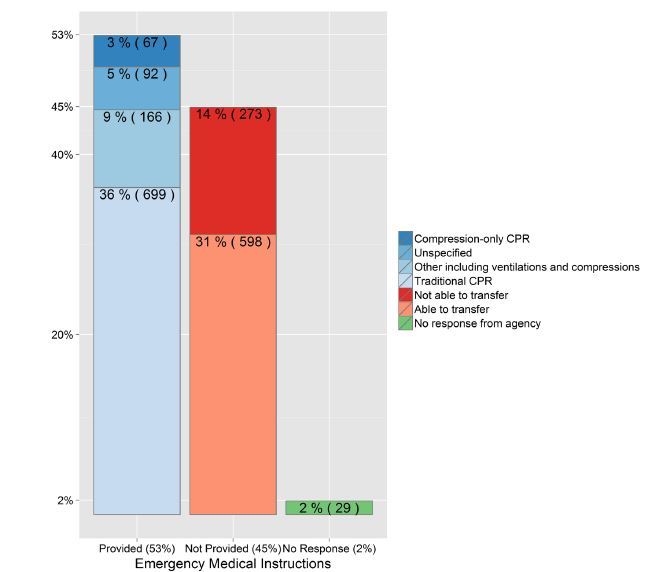
Proportion of public safety answering points providing telephone cardiopulmonary resuscitation (T-CPR) instructions and the type of CPR instructions provided to callers.

Reportedly, 881 of 1,924 (46%) responding agencies provide no T-CPR or medical instructions for medical emergencies, and 273 (14%) report that they are unable to transfer callers with medical emergencies to another facility to provide T-CPR instructions (Figure 3).

Of the 1,924 respondents, compression-only CPR instructions are reportedly provided by 3% of agencies (67/1,924), 36% (699/1,924) reported traditional CPR instructions (including chest compressions and rescue breathing), 9% (166/1,924) reported some other instructions incorporating ventilations and compressions, and 5% (92/1,924) did not specify the type of instructions (Figure 3).

### Validation

In the follow-up validation study comparing responding and non-responding agencies, the proportion of agencies that do not directly provide telephone instructions for medical emergencies did not differ significantly between responding and non-responding agencies in both the low-return subgroup (50/142 vs. 14/51, p=0.3126) and the mid-return subgroup (88/199 vs. 22/50, p=0.9775).

The proportion of agencies that use scripts or aids for the delivery of telephone instructions did not significantly differ between responding and non-responding agencies in the mid-return subgroup (89/109 vs. 24/28, p=0.7832). However, the use of scripts or aids among responding agencies is more prevalent than among non-responding agencies in the low-return subgroup (69/89 vs. 14/37, p<0.01). See supplemental material for an additional summary of the validation study (Supplement 2, Validation Study Summary).

## DISCUSSION

Bystander CPR for witnessed OHCA is believed to strongly influence survival to hospital discharge.^11^–^13^ Despite this, the rate of bystander CPR remains very low across the U.S. and likely remains a central cause of dismal survival rates in these communities.^14^,^15^ A recent AHA Scientific Advisory Statement has published specific recommendations for the provision of T-CPR instructions, including compression-only instructions for adults who suffer a sudden collapse and are not breathing normally, with the intention of improving the frequency and quality of bystander CPR being performed globally.^7^ The statement had four central recommendations: 1) 9-1-1 callers should be formally and systematically questioned to determine whether the patient may have had a cardiac arrest, and if so, CPR pre-arrival instructions should be immediately provided; 2) CPR pre-arrival instructions should be provided in a confident and assertive manner and should include straightforward chest compression–only instructions to achieve early bystander hands-only CPR for the adult who suddenly collapses; 3) individual dispatcher and organizational-level performance can be measured by using a modest set of metrics; 4) these metrics should be incorporated into an integrated quality assurance program.^7^

A detailed understanding of the current T-CPR practices of PSAPs is an essential step towards understanding the direction forward for implementing these recommendations. To our knowledge, this survey represents the first nationwide assessment of the practices of PSAPs in the U.S. regarding T-CPR instructions for cardiac arrest. Previous, smaller surveys of PSAPs with regards to T-CPR instructions were either limited to 154 dispatch centers participating in the Resuscitation Outcomes Consortium (ROC) Network^16^ and 25 EMS agencies participating in the Cardiac Arrest Registry to Enhance Survival (CARES),^17^ and provide only limited detail regarding nationwide T-CPR practices. For these studies, survey response rates of 154/154 (100%) and 21/25 (84%) were observed, respectively.

Survival rates for OHCA can vary by as much as 500% regionally in the U.S.^14^ A key aspect of EMS interventions associated with improved survival rates has been an increase in the rate of T-CPR.^18^–^20^ These survey data show that while there are many 9-1-1 centers currently providing T-CPR instructions, a substantial proportion of centers that responded to this national survey do not; this may account for a significant portion of this variability. Previous studies have shown that communities with the highest survival rates over the past several decades have consistently focused on implementing, measuring, and benchmarking this key intervention in their systems.^19^–^22^ The results of this survey suggest that there is significant potential to improve the T-CPR process through increased systematic implementation of CPR instructions, training, and quality improvement.

In addition to the need for T-CPR expansion, a closer look at the results shows significant room for improvement in the instructions provided to callers for adult OHCA. Only 3% of the responding agencies are providing instructions for compression-only CPR, which is guideline therapy for adult, out-of-hospital sudden cardiac arrest.^7^,^8^,^23^,^24^

## LIMITATIONS

As a voluntary survey of PSAPs, this study is intrinsically limited. The answers provided by agencies are assumed to be accurate representations of their practice. Our validation follow up with non-responding agencies suggests that non-responders did not differ substantially with regards to the provision of T-CPR instructions for OHCA; however, we recognize that responding agencies are likely those most involved in this topic. Consequent to the methodology used to request survey participation, this study is limited to participation from PSAPs for which e-mail addresses were procured. Although survey responses may not reflect actual practice, we would expect that 9-1-1 centers not having a structured T-CPR program to be challenged to deliver consistent guideline-based instructions.

## CONCLUSION

This large survey of PSAPs in the United States suggests that there is great variability in the implementation and measurement of the critical intervention of telephone-cardiopulmonary resuscitation instructions. There appears to be a significant opportunity to standardize and improve the delivery of telephone-CPR instructions.

## Figures and Tables

**Table 1a:** Characteristics of public safety answering points in the United States.

	N	%*
Type		
City police department	563	29
County sheriff office	381	20
State/province law enforcement	23	1
County or public agency serving one or more counties	361	19
Fire department	42	2
City and county agency	200	10
Fire and law enforcement	261	14
Joint law enforcement	18	1
Special commission	14	1
Other	14	1
Not indicated	47	2
PSAPs functioning as primary or secondary answering points		
Primary	1,199	62
Secondary	51	3
Both	659	34
Not indicated	15	1
Ambulance/EMS dispatch		
Number of PSAPs that directly dispatch ambulance/EMS	1,478	77
Quality improvement measures		
Number of PSAPs that monitor 10% or more of live calls**	448	23
Number of PSAPs that review 10% or more of recorded calls**	892	46
Number of agencies that review EMS run sheets	135	7
Number of agencies that review data from hospital records of patients transported by EMS	42	2
Number of agencies that review the time required for a caller to reach a dispatcher trained to deliver instructions	214	11
Number of agencies where EMD calls are reviewed by a supervisor, oversight committee, or peer review team	529	27
Number of agencies that complete a systematic quality review/report on a regular basis	392	20
Number of agencies with no formalized evaluation of dispatcher performance and call center services	265	14
Other measurement of dispatch service outcomes	85	4

*EMS*, emergency medical services; *PSAP*, public safety answering point; *EMD*, emergency medical dispatch

* Percentages are reported as a proportion of the total number of survey respondents (n=1,924).

** Facilities were asked to report the percentage of calls that are monitored/reviewed in 10% increments ranging from 0% to 100%.

**Table 1b:** Characteristics of public safety answering points in the United States.

	Median (Q1–Q3)	Average	Standard deviation
PSAP descriptions			
Number of dispatchers (n=1,875)	10 (6–16)	16.37	21.55
Number of annual 9-1-1 calls received (n=1,290)	12,000 (4,000–42,000)	53,000	150,015
Number of calls resulting in EMS dispatch (n=1,675)	30% (20%–50%)	37.61%	22.18%
Ambulance/EMS dispatch			
Time (seconds) to dispatch of Ambulance/EMS (n=1,120)	49 (30–60)	54.84	41.93
Time (seconds) to redirecting call to secondary PSAP if not directly dispatching EMS (n=322)	10 (5–30)	21.68	25.90
Dispatcher training			
Percentage of dispatchers providing instructions who are trained but not certified (n=1,021)	0% (0%–0%)	7.76%	23.43%
Percentage of dispatchers providing instructions who are EMD certified (n=1,021)	100% (100%–100%)	87.65%	29.47%
Quality improvement measures			
Percentage of live calls monitored by supervisory/training staff (n=448)	20% (10%–50%)	32.95%	28.41%
Percentage of recorded calls reviewed by supervisory/training staff (n=892)	20% (10%–50%)	33.71%	28.05%

*PSAP*, public safety answering point; *EMS*, emergency medical services; *EMD*, emergency medical dispatch

**Table 2:** Structured script and guideline-based protocol use at public safety answering points that provide instructions for medical emergencies.

Script/guideline use	n	%	Type of script/aid	n	%
Structured script	834	83	A manual system (e.g. printed cards)	507	61
Written guidelines	138	14	A computer-based system	318	39
No script or guidelines	30	3			
Total	1,002	100	Total	825	100
